# Expression of Concern: Pneumonia remains a leading public health problem among under-five children in peri-urban areas of north-eastern Ethiopia

**DOI:** 10.1371/journal.pone.0334271

**Published:** 2025-10-10

**Authors:** The PLOS One Editors

The *PLOS One* Editors issue this Expression of Concern to alert readers that this article [1] was identified as one of a series of submissions for which we have concerns about authorship and adherence to the journal’s first and second publication criteria. In addition, the article’s claim that pneumonia remains a leading public health problem among under-five children in peri-urban areas of north-eastern Ethiopia is not supported, as this study does not compare the prevalence of pneumonia with that of other public health problems.

During editorial follow-up, the *PLOS One* Editors also noticed the following discrepancies in the article’s ethics approval and proposal that were provided to the journal post-publication:

- In the research proposal document, the final sample size of 560 was calculated with a 10% anticipated non-response rate, but the published article states that a 5% non-response rate was anticipated.
- The research proposal cited “539 estimated participants” multiple times despite the sample size being 560. This value matches the final number of respondents reported in the article [1].

First author AK responded that the proposal submitted to the department included sample size determination followed by a reduction formula and a 10% non-response rate as a conservative estimate, but that this calculation was not correct, and that following further discussion and literature review, the authors revised it to a 5% non-response rate. AK also responded that the sample size in one section of the research proposal was mistakenly reported as 539 due to a typographical error.

The *PLOS One* Editors do not consider these issues fully resolved in light of the authors’ responses. We regret that these issues were not addressed prior to the article’s publication. Readers are advised to interpret this article’s [1] results with caution.

Author AK also provided the following additional information about the subject selection process: The households within each *kebele* that had at least one child under five were identified with the assistance of local health extension workers. Systematic random sampling with an interval of six was used to select households in each *kebele* and one child was selected per household. In households with more than one child under five, only one child was included to maintain statistical independence. A simple lottery method was used for selection; the names or initials of each eligible child were written on separate pieces of paper, placed into a container, and one was drawn at random by the data collector in the presence of the mother/caregiver.

The article’s ethics statement does not report an ethics approval number or date. The first sentence of the Ethical consideration section is updated to the following: The ethical approval letter was obtained from the Institutional Ethical Review Committee of the College of Medicine and Health Sciences of Wollo University on January 30, 2019, reference number 506/02/11.

In addition, contrary to the article’s Data Availability statement, the original raw data files supporting the article’s results were not provided with the article. The Data Availability statement is updated to: All relevant data are available from https://doi.org/10.7910/DVN/R4GZ7F.
