# Supplementary material for: Pneumonia remains a leading public health problem among under-five children in peri-urban areas of north-eastern Ethiopia
Source: PLoS One. 2020 Sep 11;15(9):e0235818. doi: 10.1371/journal.pone.0235818 (PMC7485761; doi:10.1371/journal.pone.0235818)
Supplement: S1 File — (DOCX) [file pone.0235818.s001.docx]

**S1 Household survey questionnaire, English version**

**Participant ID No___________ Kebele ____ Interviewer name_________________________ Date of interview____________ Starting time _____________Ending time ___________**

**Please encircle the correct answer which to indicate the number and write a correct number example age put in a year.**

**Part I: Socio-demographic characteristics**

| **S.No** | **Questions** | **Answer** | **Skip** |
| --- | --- | --- | --- |
| 101 | Age of the mother/caregiver(years) | ______________ |  |
| 102 | Age of the child (months) | _________ |  |
| 103 | Sex of the child | 1= Male  2 =Female |  |
| 104 | Religion of mother/caregiver | 1=Orthodox  2=Muslim |  |
| 105 | Level of education of mother/caregiver | 1=Unable to read & write  2=Read and write  3=Primary level  4=Secondary level  5=Diploma and above |  |
| 106 | Maternal occupation | 1=Housewife  2=Civil servant  3=Daily laborer  4=Merchant  5=Other_________ |  |
| 107 | Marital status of mother/caregiver | 1=Married  2=Unmarried |  |
| 108 | If you have a husband, what is the educational level of your husband? | 1=Unable to read & write  2=Read and write  2=Primary level  3=Secondary level  4=Diploma and above |  |
| 109 | If you have a husband, what is the occupational status of your husband? | 1=Unemployed  2=Farmer  3=Daily laborer  4=Merchant  5=Civil servant |  |
| 110 | Total number of family size | _______________ |  |

**Urban wealth questions**

| 111  112  113  114  115  116  117 | What is the source of your drinking water? (more than one answer is possible )  1.houseline water Yes ... 1 No ... 0  2. pull and push/sway common water Yes ... 1 No ... 0  3. bono water Yes ... 1 No ... 0  4.protective pond water Yes ... 1 No ... 0  5.un protective pond water Yes ... 1 No ... 0  6.stream water Yes ... 1 No ... 0  7. if others list…….. | |  |
| --- | --- | --- | --- |
| 118 | What type of toilet do you use?  1. Water flush Yes ... 1 No ... 0  2. Traditional toilet Yes ... 1 No ... 0  3. Ventilated improved pit latrine Yes ... 1 No ... 0  4. Open field Yes ... 1 No ... 0  5. Others (list)……. | |  |
| 119 | Who is the owner of your living house? 1. My own 0. rent house | |  |
| 120 | Does your living house have dividing class? Yes ... 1 No ... 0 | |  |
| 121 | Do you have a separated bedroom? Yes ... 1 No ... 0 | |  |
| 122 | Do you have a separated kitchen? Yes ... 1 No ... 0 | |  |
| 123  124  125  126  127 | From which material your house floor is made? (more than one answer is possible )  1. Natural ground Yes ... 1 No ... 0  2. Muck/smooth by cows faces Yes ... 1 No ... 0  3. Wood Yes ... 1 No ... 0  4. Cement Yes ... 1 No ... 0  5. if others list …….. | |  |
| 128 | From which material your house roof is made?  0. Grass/ leaf 1. corrugated iron | |  |
| 129  130  131  132  133 | From which material your house wall is made? (more than one answer is possible )  1. Wood but not have mod Yes ... 1 No ... 0  2. Wood with mod Yes ... 1 No ... 0  3. Wood and cement Yes ... 1 No ... 0  4. Blocket Yes ... 1 No ... 0  5. if others list…. | |  |
| 134  135  136  137  138  139 | What is your energy source for food cooking? (more than one answer is possible )  1. Electric city system Yes ... 1 No ... 0  2. Gas /kerosene Yes ... 1 No ... 0  3. Wood /leaf Yes ... 1 No ... 0  4. Charcoal Yes ... 1 No ... 0  5. Animal feces Yes ... 1 No ... 0  6. If another list… | |  |
| 140  141  142  143  144  145  146  147  148  149  150  151  152  153 | Among the following materials, which one do you own? (more than one answer is possible )  1. Radio Yes ... 1 No ... 0  2. Television Yes ... 1 No ... 0  3. House phone Yes ... 1 No ... 0  4. Fridge Yes ... 1 No ... 0  5. Chair Yes ... 1 No ... 0  6. Table Yes ... 1 No ... 0  7. Bed and mattress which made from cotton spring Yes ... 1 No ... 0  8. Mobile Yes ... 1 No ... 0  9. Cycle Yes ... 1 No ... 0  10. Motorcycle Yes ... 1 No ... 0  11. Horse’s cart Yes ... 1 No ... 0  12. Bajaj/car Yes ... 1 No ... 0  13.Bank book Yes ... 1 No ... 0  14. If another list…. | |  |
|  | **Rural wealth questions** | |  |
|  |  | |  |
| 154  155  156  157  158  159  160  161 | | Watch Yes ... 1 No ... 0  Sofa Yes ... 1 No ... 0  Chair Yes ... 1 No ... 0  Table Yes ... 1 No ... 0  Bed and mattress which made from cotton spring Yes ... 1 No ... 0  Horse’s Cart Yes ... 1 No ... 0  If others specify...  Do you have your own farm for the purpose of agriculture/cropping? Yes ... 1 No ...0 |  |
| 162  163  164  165  166  167  168 | | From the following household animal do you have? (can answer more than one)  1. Ox/ cow Yes ...1 No ... 0  2. Horse/donkey/ mule Yes ... 1 No ... 0  3. Goat Yes ... 1 No ... 0  4. Sheep Yes ... 1 No ... 0  5. Hen Yes ... 1 No ... 0  6. Beehive Yes ...1 No ... 0  7. Others................... |  |

**Part II: Housing and Environmental factors (observation)**

| **S.No** | **Questions** | **Answer** | **skip** |
| --- | --- | --- | --- |
| 201 | House ownership | 1 rent 2. Private ownership |  |
| 202 | Type of roof of the house  (observation) | 1= Corrugated Iron Sheet  2=Tukul/thatched  3=Other(Specify)___________ |  |
| 203 | Type of the wall (observation) | 1=Wood with mud  2=Stone and mud/cement/bricks |  |
| 204 | Type of house floor (observation) | 1=Earth /Soil  2=Cement/ Ceramic |  |
| 205 | Does the kitchen have a window? | 0=No  1=Yes |  |
| 206 | Number of windows in the main house | _________________ |  |
| 207 | Do you have a separate room used as Kitchen? | 0=No  1=Yes |  |
| 208 | Where do you cook? | 1= Kitchen  2= Living room |  |
| 209 | What type of fuel used for cooking? | 1=Electricity  2=Domestic(Wood/Charcoal/kerosene) |  |
| 210 | Where do you put the child during cooking? | 1=Outside of the cooking house  2=Caring mothers back |  |
| 211 | Is there any cigarette smoker? | 0=No  1=Yes |  |
| 212 | Number of rooms of the house(observation) | ______________________ |  |
| 213 | Total house area | _____________________ |  |

**Part III. Nutritional factors and immunization status**

| **S.No** | **Questions** | **Answer** | **Skip** |
| --- | --- | --- | --- |
| 301 | Vitamin A supplementation | 0=No  1=Yes |  |
| 302 | Zinc Supplementation | 0=No  1=Yes |  |
| 303 | Do the child take pneumococcal conjugate vaccine(PCV) | 0=No  1=Yes |  |
| 303 | Vaccination Status(see the card) | 1=Fully vaccinated  2=Incomplete(Up to date ,Partial vaccinated and Unvaccinated) |  |
| 304 | What do you feed your child during the first 6 months? | 1=Exclusive breastfeeding  2=Mixed Breastfeeding |  |
| 305 | How many months do you breast feed? | _____________________ |  |

**Part IV. Preexisting medical or Co-morbid conditions**

|  | **Questions** | **Answer** | **Skip** |
| --- | --- | --- | --- |
| 401 | Child history of ARI | 0=No  1=Yes |  |
| 402 | Family history of ARI | 0=No  1=Yes |  |
| 403 | Child history of Congenital Heart Disease | 0=No  1=Yes |  |
| 404 | Child history of HIV/AIDS | 0=No  1=Yes |  |
| 405 | Child history of Tuberculosis (TB) | 0=No  1=Yes |  |
| 406 | Child history of Asthma | 0=No  1=Yes |  |
| 407 | Acute malnutrition using MUAC measurement | 1__________ 2___________ |  |

**Part V Pneumonia sign & symptoms as well as physical examination findings using measurement device**

| **S.NO** | **Question** | **Answer** | **skip** |
| --- | --- | --- | --- |
| 501 | Cough | 0=No  1=Yes |  |
| 502 | Fast breathing | 0=No  1=Yes |  |
| 503 | Chest tightness/indrawing | 0=No  1=Yes |  |
| 504 | Fever(chills) | 0=No  1=Yes |  |
| 505 | Pneumonia | 1= No  2= Yes |  |

**Thank you for your valuable information and participation!!!**
