# Supplementary material for: Pneumonia remains a leading public health problem among under-five children in peri-urban areas of north-eastern Ethiopia
Source: PLoS One. 2020 Sep 11;15(9):e0235818. doi: 10.1371/journal.pone.0235818 (PMC7485761; doi:10.1371/journal.pone.0235818)
Supplement: S2 File — (DOCX) [file pone.0235818.s002.docx]

**አማርኛ መጠይቅ**

የተሳታፊ መለያ ቁጥር _____ቀበሌ _____የቃለ

መጠይቅ አድራጊው ስም _______________ቀን_____________

የተጀመረበት-----------------ሰዓት_____________________የተጠናቀቀበት ሰዓት____

ክፍል 1፡ **የሶሽዮ-ዲሞግራፊ መረጃ መጠይቅ**

| ተ.ቁ | ጥያቄ | አማራጭ | እለፍ |
| --- | --- | --- | --- |
| 101 | የሕፃኑ/ኗ እናት/አሳዳጊ ዕድሜ በአመት | ----------- |  |
| 102 | የሕፃኑ/ኗ ዕድሜ | ------------- |  |
| 103 | የሕፃኑ/ኗ ፆታ | 1. ወንድ 2. ሴት |  |
| 104 | የቤተሰብ ወይም ያአሳዳጊ ሀይማኖት | 1. ኦርቶዶክስ 2. ሙስሊም 3. ፕሮቴስታንት 4. ካቶሊክ 5. ሌላ ካለ ይገለፅ…………………… |  |
| 105 | የሕፃኑ/ኗ እናት/አሳዳጊ የጋብቻ ሁኔታ | 1. ያላገባች 2. ያገባች 3. ተለያይተው የሚኖሩ 4. የፈታች 5. ባል የሞተባት |  |
| 106 | የሕፃኑ/ኗ እናት/አሳዳጊ የትምህርት ደረጃ | 1. ማንበብና መፃፍ የማትችል 2. ማንበብ እና መፃፍ የምትችል 3. አንደኛ ደረጃ 4. ሁለተኛ ደረጃ 5. ኮሌጂ እና ዩንቨርስቲ |  |
| 107 | የሕፃኑ/ኗ እናት/አሳዳጊ የስራ ሁኔታ | 1. ስራ የሌላት 2. የመንግስት ሰራተኛ 3. የቀን ሰራተኛ 4. ነጋዴ 5. ሌላ ካለ ይገለፅ………………………… |  |
| 108 | ባለቤት ካለዎት የባለቤትዎ የትምህርት ደረጃ | 1. ማንበብና መፃፍ የማይችል 2. ማንበብ እና መፃፍ የሚችል 3. አንደኛ ደረጃ 4. ሁለተኛ ደረጃ 5. ዩንቨርስቲ እና ኮሌጂ |  |
| 109 | ባለቤት ካለዎት የባለቤትዎ የስራ ሁኔታ | 1. ስራ የሌለው 2. የመንግስት ሰራተኛ 3. የቀን ሰራተኛ 4. ነጋዴ 5. ሌላ ካለ ይገለፅ________________________ |  |
| 110 | የቤተሰብ ብዛት | ________________________________________ |  |

**የከተማ ሐብት ሁኔታ ጠቋሚ መረጃ**

|  | የምትጠቀሙት ዉሃ መገኛው ከየት ነዉ? (ከአንድ በላይ መመለስ ይቻላል) |  |
| --- | --- | --- |
| 111 | 1. በቤት ዉስጥ ያለ የመስመር ዉሃ አወ …..1 የለም …..0 |  |
| 112 | 2. በእጅ የሚነቀነቅ የጋራ ዉሃ አወ …..1 የለም….. 0 |  |
| 113 | 3. ቦኖ ዉሃ አወ …..1 የለም…..0 |  |
| 114 | 4. የተከለለ የጉድጋድ ዉሃ አወ …..1 የለም….. 0 |  |
| 115 | 5 ያልተከለለ የጉድጋድ ዉሃ አወ …..1 የለም….. 0 |  |
| 116 | 6. የምንጭ /የወራጅ/ ኩሬ የግድብ ዉሃ አወ …..1 የለም….. 0 |  |
| 117 | 7. ሌላ ካለ ይጥቀሱ ----- |  |
| 118 | የምትጠቀሙበት መጸዳጃ ቤት ምን አይነት ነዉ? |  |
|  | 1. በዉሃ የሚሰራ ሽንት ቤት  2. የአየር ማስወጫ ቱቦ ያለዉ ሽንት ቤት  3. ባህላዊ ሽንት ቤት  4. ሜዳ ላይ  5. ሌላ ካለ ይጥቀሱ ------- |  |
| 119 | የምትኖሩበት ቤት ባለቤቱ ማን ነዉ? 1. የግልዎ 2. ተከራይተው |  |
| 120 | መኖሪያ ቤታችሁ የተለያየ አገልግሎት እንድሰጥ የተከፋፈለ ነውን? አወ…..1 የለም…..0 |  |
| 121 | የተለየ የምኝታ ክፍል ለብቻ አለ ወይ? አወ….1 የለም….0 |  |
| 122 | የተለየ የምግብ ማብሰያ /ኩሽና ክፍል አለ ወይ? አወ…..1 የለም…..0 |  |
| 123 | የቤቱ ወለል ከምንድን ነዉ የተሰራዉ? (ከአንድ በላይ መመለስ ይቻላል) |  |
|  | 1. የተፈጥሮ መሬት /ወለሉ ምንም ነገር የሌለዉ አወ …..1 የለም….. 0 |  |
| 124 | 2. በከብቶች እበት የተሰራ /የተለቀለቀ ወለል አወ …..1 የለም….. 0 |  |
| 125 | 3. በእንጨት የተሰራ ወለል አወ …..1 የለም….. 0 |  |
| 126 | 4. በሲሚነቶ የተሰራ ወለል አወ …..1 የለም….. 0 |  |
| 127 | 5. ሌላ ካለ ይጥቀሱ….. |  |
| 128 | የቤቱ ጣራ ከምድን ነዉ የተሰራዉ? 1. ከሳር/ከቅጠል 2. ከቆርቆሮ |  |
| 129 | የቤቱ ግድግዳ ከምንድን ነዉ የተሰራዉ? (ከአንድ በላይ መመለስ ይቻላል) |  |
|  | 1. ከእንጨት ሁኖ ጭቃ የሌለዉ አወ …..1 የለም….. 0 |  |
| 130 | 2. ከእንጨት እና ከጭቃ አወ …..1 የለም….. 0 |  |
| 131 | 3. ከእንጨት እና ከሲሚነቶ አወ …..1 የለም …..0 |  |
| 132 | 4. ከብሎኬት የተሰራ አወ…..1 የለም….. 0 |  |
| 133 | 5. ሌላ ካለ ይጥቀሱ…… |  |
| 134 | ለምግብ ማብሰያነት የምትጠቀሙበት የሀይል ምንጭ Uንድን ነዉ? (ከአንድ በላይ መመለስ ይቻላል) |  |
|  | 1. ኤሌክትሪክ አወ… 1 የለም….. 0 |  |
| 135 | 2. ነጭ ጋዝ አወ… 1 የለም….. 0 |  |
| 136 | 3. እንጨት /ቅጠል አወ… 1 የለም….. 0 |  |
| 137 | 4. ከሰል አወ… 1 የለም….. 0 |  |
| 138  139 | 5. ኩበት/በጠጥ አወ… 1 የለም….. 0  6. ሌላ ካለ ይጥቀሱ…… |  |
|  | ክዚህ በታች ከተዘረዘሩት ንብረቶች በቤታችሁ ዉስጥ ያላችሁ የቱ ነዉ? (ከአንድ በላይ መመለስ ይቻላል) |  |
| 140 | 1. ሬደዮ አወ…….1 የለም……..0 |  |
| 141 | 2. ቴሌቪዥን አወ……1 የለም……..0 |  |
| 142 | 3. የቤት ስልክ አወ…….1 የለም…….0 |  |
| 143 | 4. ፍሪጅ አወ……1 የለም..……0 |  |
| 144 | 5. ወንበር አወ…….1 የለም…….0 |  |
| 145 | 6. ጠረንጴዛ አወ…….1 የለም…….0 |  |
| 146 | 7. የጥጥ/የእስቦንጅ / አስፕሪነግ ፍራሽ ያለዉ አልጋ አወ……1 የለም….0 |  |
| 147 | 8. ሞባይል ስልክ አወ………1 የለም…….0 |  |
| 148 | 9. ሳይክል አወ………1 የለም..…..0 |  |
| 149 | 10. ሞተርሳይክል አወ………1 የለም…….0 |  |
| 150 | 11. የፈረስጋሪ አወ………1 የለም……0 |  |
| 151 | 12. ባጃጅ/መኪና አወ………1 የለም……0 |  |
| 152 | 13. የባንክ ቡክ አወ………1 የለም……0 |  |
| 153 | 14. ሌላ ካለ ይጥቀሱ…… |  |

**የገጠር ሐብት ሁኔታ ጠቋሚ መረጃ**

| 154 | የግላችሁ የሆነ ለምርት/እርሻ የሚሆን መሬት አላችሁ ወይ ; አወ………1 የለም….…0 |  |
| --- | --- | --- |
|  | ከሚከተሉት የቤት እንሰሳት ዉሰጥ የትኛው አላችሁ? (ከአንድ በላይ መመለስ ይቻላል) |  |
| 155 | 1. በሬ፣ላም አወ………1 የለም…..…0 |  |
| 154 | 1. ፈረስ/አህያ፣በቅሎ አወ………1 የለም……..0 |  |
| 155 | 1. ፍየል አወ………1 የለም……..0 |  |
| 156 | 1. በግ አወ………1 የለም…….0 |  |
| 157 | 1. ዶሮ አወ………1 የለም……..0 |  |
| 158 | 1. የንብ ቀፎ አወ………1 የለም……..0 |  |
| 159 | 1. ሌላ ካለ ይጥቀሱ…… |  |

**ክፍል 2: የቤት እና የአካባቢ ሁኔታ**

| ተ.ቁ | ጥያቄ | ምላሽ | እለፍ |
| --- | --- | --- | --- |
| 201 | የቤቱ ጣራ አይነት(በማየት የሚሞላ) | 1=ቆርቆሮ  2. ሳር  3. ሌላ(ይጠቀስ)_________________ |  |
| 202 | የግድግዳው አይነት(በማየት የሚሞላ) | 1=እንጨትና ጭቃ  2= ድንጋይና ጭቃ  3=ድንጋይና ስሚንቶ  4=ብሎኬት/ከጡብ |  |
| 203 | የቤቱን ወለል አይነት(በማየት የሚሞላ) | 1=አፈር  2= ስሚንቶ/ሴራሚክ  3=ጣዉላ  4=ሌላ(ይጠቀስ)_________________ |  |
| 204 | ቤቱ ስንት መስኮት አለው(በማየት የሚሞላ) | ______________________ |  |
| 205 | ለምግብ ማብሰያ የሚጠቀሙበት የተለየ ክፍል  አላችዉ ? (በማየት ይረጋገጥ) | 0=የለም  1=አለ |  |
| 206 | የምግብ ማብሰያ ቤቱ መስኮት አለው?(በማየት ይረጋገጥ) | 0=የለም  1=አለ |  |
| 207 | ምግብ የት ታበስላላችሁ? | 1=ምግብ ማብሰያ ክፍል  2. በቤት ውስጥ |  |
| 208 | በቤት ውስጥ የሚጠቀሙት የማብሰያ ሃይል ምንጭ ምንድን ነው? | 1=ኤሌክትሪክ  2=ባህላዊ (ነጭ ጋዝ/ከሰል /እንጨት) |  |
| 209 | ምግብ በምታበስይ ጊዜ ህጻኑን የት ታስቀምጭዋለሽ | 1=ከቤት ውጭ  2=አዝለዋለሁ |  |
| 210 | የቤት እንስሳዎች (ዶሮ፤ በግ፤ ፍየል ወዘተ)ካሏችሁ የት ነዉ የሚያድሩት? | 1=ዉጭ ላይ/በረት ላይ  2=የተለየ ክፍል ማደሪያ አላቸዉ  3=ከቤተሰቡ ጋር በአንድ ክፍል |  |
| 211 | ቤት ውስጥ የሚያጨስ ስው አለ? | 0=የለም  1=አለ |  |
| 212 | ቤትዎ ስንት ክፍል አለው? | ___________________________ |  |
| 213 | መኖርያ ቤትዎ ምን ያህል ይሰፋል? (M^2^) | ______________________________ | |

**ክፍል 3.የአመጋገብ እና የክትባት ሁኔታ**

| ተ.ቁ | ጥያቄ | ምላሽ | እለፍ |
| --- | --- | --- | --- |
| 301 | ሕፃኑ/ኗ ቫይታሚን ኤ ወስዷል/ለች? | 0. አልወሰደም/ችም  1=አዎ |  |
| 302 | ሕፃኑ/ኗ የዚንክ እንክብል ወስዷል/ለች? | 0. አልወሰደም/ችም  1=አዎ |  |
| 303 | ሕፃኑ/ኗ የሳንባ ምች ክትባት ወስዷል/ለች? | 0. አልወሰደም/ችም  1=አዎ |  |
| 304 | ሕፃኑ/ኗ . ሌሎች ክትባትች ወስዷል/ለች?(በማየት ይረጋገጥ) | 1=ከትባቱን የጨረሰ  2=እየተከተበ ያለ  3=በከፊል የተከተበ  4=ምንም ያልተከተበ |  |
| 305 | ከተወለደ እስከ 6 ወር ህጻኑን ምን መገብሽው? | 1=የእናት ጡት ወተት ብቻ  2=የእናት ጡት ወተት እና ተጨማሪ ምግብ |  |
| 305 | ልጅሽን ለምን ያህል ወራት ጡት አጠባሽው? | ____________________________________ |  |

**ክፍል 4.የቀደመ የጤና ችግር**

| ተ.ቁ | ጥያቄ | ምላሽ | | እለፍ |
| --- | --- | --- | --- | --- |
|  | ከታች ከተገለጹት በሃኪም የተረጋገጠ የጤና ችግር አለብዎት? | 0 = የለም | 1 = አዎ |  |
| 401 | ባለፉት ሁለት ሳምንታት የመተንፈሻ አካላት በሽታ(ለሕፃኑ/ኗ) | 0 = የለም | 1 = አዎ |  |
| 402 | ባለፉት ሁለት ሳምንታት የመተንፈሻ አካላት በሽታ(ለቤተሰቡ) | 0 = የለም | 1 = አዎ |  |
| 403 | ልብህ መም(ለሕፃኑ/ኗ) | 0 = የለም | 1 = አዎ |  |
| 404 | ኤች አይ ቫ/ኤድስ | 0 = የለም | 1 = አዎ |  |
| 405 | የሳንባ ትቪ(ለሕፃኑ/ኗ) | 0 = የለም | 1 = አዎ |  |
| 406 | አስም (ለሕፃኑ/ኗ) | 0 = የለም | 1 = አዎ |  |
| 407 | የተመጣጠነ ምግብ እጥረት (የክንድ ልኬት) | ልኬት 1 ____________ | ልኬት 2_____ |  |

**ክፍል 5. የሳንባ ምች ህመም ምልክቶች**

| ተ.ቁ | ጥቄ | | ምላሽ | | እለፍ |
| --- | --- | --- | --- | --- | --- |
| ከተዘረዘሩት ውስጥ የትኞቹ ህመም ምልክቶች በህጻኑ/ኗ ላይ ይታያል? በመጠየቅ + በማየት /በመመርመር የሚሞላ | | | | |  |
| 501 | ሳል | 0 = የለም | | 1 =አወ/አለ |  |
| 502 | ለመተንፈስ መቸገር | 0 = የለም | | 1 =አወ/አለ |  |
| 503 | የደረት ውጋት | 0 = የለም | | 1 =አወ/አለ |  |
| 504 | ትኩሳ (ማንቀጥቀጥ/ብርድ ብርድ ማለት) | 0 = የለም | | 1 =አወ/አለ |  |

**ስለተሳትፎዎ እናመሰግናለን !!!**
