# Supplementary material for: Pneumonia remains a leading public health problem among under-five children in peri-urban areas of north-eastern Ethiopia
Source: PLoS One. 2020 Sep 11;15(9):e0235818. doi: 10.1371/journal.pone.0235818 (PMC7485761; doi:10.1371/journal.pone.0235818)
Supplement: Attachment — Submitted filename: Response to reviwers.docx [file pone.0235818.s003.docx]

**Rebuttal letter**

**Response to the Journal Requirements Questions**

**Question #1: Please ensure that your manuscript meets PLOS ONE's style requirements, including those for file naming.**

**Response:** Thank you for this remark. We re-formatted the revised manuscript using the PLoS ONE format guidelines. The whole content of the manuscript, including the abstract, introduction, methods, discussion and reference are formatted using the guidelines (**please see the revised version for each section**).

**Question #2.** Please include additional information regarding the survey or questionnaire used in the study and ensure that you have provided sufficient details that others could replicate the analyses. For instance, if you developed a questionnaire as part of this study and it is not under a copyright more restrictive than CC-BY, please include a copy, in both the original language and English, as Supporting Information.

**Response: We provided the questionnaire in original language and English version as supporting information S1 and S2.**

**Question #3.** - Please provide additional details regarding participant consent. In the ethics statement in the Methods and online submission information, please ensure that you have specified how verbal consent was documented and witnessed.

**Response: We provided all the ethical consideration sub-heading (please see page 10 from lines 245 to 250.**

**Question#4.** Please ensure that you refer to Figure 2 and 4 in your text as, if accepted, production will need this reference to link the reader to the figure.

**Response: We did the citation of Fig 1, 2 and 3 in the texts. One figure is reduced during the revision. Then the total figure becomes 3 not 4.**

**Line by line response to reviewers**

**Reviewer # 1**

**Question #1.** Table 1 is missing. The serial starts from Table 2. However table 1 is referenced in the legend. To attach the table 1.

Response: Thank you for this comment and we included Table 1.

**Question #2.** Justify how weaning period of 12 or more months is taken as reference category with WHO recommendations of weaning at 6 months of age.

**Response:** Sorry for the confusion we did. We studied about the total months for breast feeding as less than 12 months and 12 months and above. Then using the 12 months as a reference category for analysis (See Table 4).

**Reviewer #2**

Reviewer #2: The manuscript entitle ¨Prevalence and associated cactors of pneumonia among children under five in peri-urban areas of Dessie City, northeastern Ethiopia: A community-based cross sectional study¨ described by Kelleb A. et al., is very well written, interesting with values information about pneumonia. It is very difficult to perform a field study, moreover in Kebeles population. The authors accumulated an impressive data that should be used for others studies and diseases. The questionnaire and analyses were intensely performed and the results and conclusions included all comments of the observational study. In my opinion, the authors should develop more the subject about vaccination.

Question #1 It is a relevant thematic in the pneumonia domain and some information are missed, such us, the definition about fully vaccinated¨ and ¨not fully vaccinated. I am not aware about the vaccination system in Ethiopia.

Response: Thank you for this pertinent comment. In Ethiopia, fully vaccination means the child received BCG and OPV0 at birth, pentavalent 1 (DPT-hib, he-b), OPV1, and PCV1 at 6 weeks, pentavalent 2, OPV2, PCV2 at 10 weeks, pentavalant 3, and PCV3 at 14^th^ weeks and measles at 9 months, whereas incomplete vaccination includes those children in the status of up-to-date, partial and totally unvaccinated (See in page 8 from lines 186 to 190)

**Reviewer #3**

Reviewer #3: The study is about children with clinical signs of pneumonia not confirmed cases of pneumonia since no microbiological or radiological diagnosis done.

Question #1. Table 1,2,3,4,5 and 6 the first line and column 2 it is write (Pnrumonia N = 539) this is confusing, 539 is it the number of participant or number of children with pneumonia ? What are the meaning of yes and no of this column? What about presence and absence of pneumonia knowing that 92 children had pneumonia?

***Response:***

Thank you for these important questions. We made a correction for the columns of each table and we deleted N = 539. However, Yes or No in the column is about to indicate the number of pneumonia and no pneumonia among children under-five. Absence of pneumonia means “No” and presence of pneumonia means “Yes”. Please see the revised Tables 1-5.

**Question # 2. Table 4 : the pneumonia vaccine and age at which it was administered was not mentioned in the study and out of 492 (75+417) children vaccinated against pneumonia 417 (yes) have pneumonia. Vaccination and occurrence of pneumonia is an important point that should be discussed?**

**Response**: In this study, a total of 492 children took the pneumonia vaccine at their respective age, but from 492 only 75 children were developing pneumonia. This may due to vaccines losing their potency because of poor vaccine stock management, poor vaccine handling and storage at storage centers even if they were potent on arrival (please see the revised version in page 14 from lines 349 to 352).

**Question # 3. The word *Kebele* have been used many times without giving the meaning.**

**Response**: We defined kebele and please see the revised version page 5 from lines 121 to 122.

**Question #4. Line 314-315 comparison of pneumonia prevalence of localized peri-urban area of Ethiopia to that of National level of Brazil done. What is the aim of this?**

**Response**: We thank for this important question, and we accept the comment and deleted the comparison since our study setting in a small area where the Brazil is national survey.

**We would like to thank the reviewers and editors for evaluating our manuscript. We have tried to address all the concerns in a proper way and believe that our paper has been improved considerably. We would be happy to make further corrections if necessary and look forward to hearing from you all soon.**

I hope that the revised manuscript is accepted for publication in PLoS ONE.

Sincerely yours,

Metadel Adane (PhD)
